# Dark side illuminated: imaging of *Toxoplasma gondii* through the decades

**DOI:** 10.1186/1756-3305-6-334

**Published:** 2013-11-22

**Authors:** Kathryn E McGovern, Emma H Wilson

**Affiliations:** 1 School of Medicine, Division of Biomedical Sciences, University of California, Riverside, CA 92521-0129, USA

**Keywords:** Microscopy, *Toxoplasma gondii*

## Abstract

In the more than 100 years since its discovery, our knowledge of *Toxoplasma* biology has improved enormously. The evolution of molecular biology, immunology and genomics has had profound influences on our understanding of this ubiquitous bug. However, it could be argued that in science today the adage "seeing is believing" has never been truer. Images are highly influential and in the time since the first description of *T. gondii*, advances in microscopy and imaging technology have been and continue to be dramatic. In this review we recount the discovery of *T. gondii* and the contribution of imaging techniques to elucidating its life cycle, biology and the immune response of its host.

## Review

### Introduction

When Charles Nicolle, Louis Manceaux and Alfonso Splendore first described *Toxoplasma gondii* in 1908, their depiction of the parasite was similar and very detailed [1,2]. Both papers, presented days apart, describe *T. gondii* as a parasite found both inside and outside of nucleated cells, never in red blood cells, having a rounded or piriform shape, and with a length of 5–8 μm. Splendore describes the wasting exhibited by all the rabbits he was studying before they succumbed to infection. He goes on to describe the hypertrophied and discolored spleen, the enlarged liver and lymph nodes and the ulcerated small intestine. He even describes, in addition to the parasite’s commonly observed "kidney-shaped form", the presence of cysts, 8–40 μm in diameter. Nicolle and Manceaux focus their efforts on describing *T. gondii’s* morphology and systematically recount in which types of tissues the parasite is found in the gundis they were studying. Both papers underscore *T. gondii’s* similarity to *Leishmania*, so much so that Nicolle and Manceaux initially proposed to call their new parasite, *Leishmania gondii*[1,2].

It is now known that *Toxoplasma* is an obligate intracellular parasite that can invade any nucleated cell in any warm-blooded animal. The prevalence rate of this parasite is phenomenal with recent estimates at just under 10% in China, between 15-30% in the US and UK and up to 80% in areas of Europe and South America [3]. *T. gondii* is categorized into three main clonal lineages: type I (the most virulent), type II (the most common in the U.S. and Europe), and type III (the least virulent). Infection stimulates a proinflammatory immune response with systemic parasitemia contained within one to two weeks. The host remains infected for life and a continuous T cell response is required to prevent reactivation of *Toxoplasma* cysts. Severe pathology therefore manifests in the immune compromised, most commonly observed as Toxoplasmic encephalitis.

Despite the detailed description of *Toxoplasma* in the original papers and despite the fact that photographic recordings of magnified images were being made using the daguerreotype method since 1840 [4], neither of the manuscripts published by Nicolle and Manceaux or Splendore were accompanied by images of this newly discovered parasite. In order to fully understand either paper, the reader had to already be familiar with *Leishmania*. We now know the differences between these parasites are substantial. In the 100 years since Nicolle and Manceaux made the initial description, light microscopy, fluorescence and electron microscopy have all been invented and applied to the study of *T. gondii*. In addition, genetic and molecular approaches have allowed us to identify and tag multiple proteins within a cell. In this review, we examine the contribution that microscopic images have made to our knowledge of *Toxoplasma’s* structure, behavior and biology.

### Light microscopy

The most commonly used and basic technique is brightfield light microscopy, where preserved slices of tissue are mounted onto slides and stained with acidophilic, basophilic, or reactive dyes to enhance various features of the cells.

**Figure 1:**
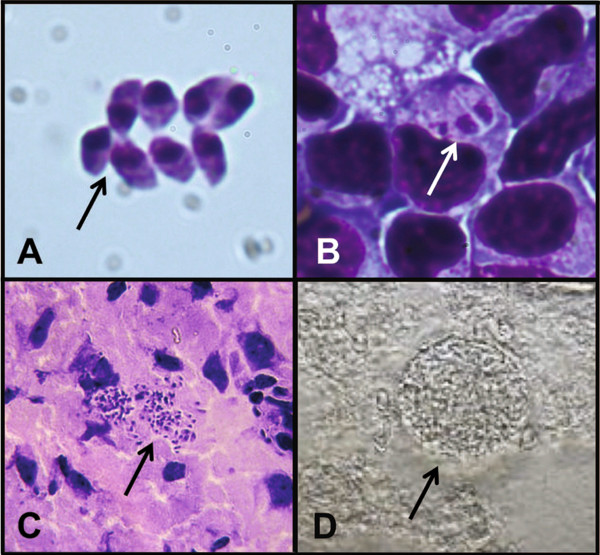
***T. gondii *stages visualized by light microscopy. A**. Extracellular tachyzoites from the peritoneum of a mouse seven days post infection, stained with H&E. (63x) **B**. Replicating parasites inside a monocyte, stained with H&E. (63x) **C**. Bradyzoites found in the brain of a chronically infected mouse, stained with H&E. (40x) **D**. Unstained cyst from brain homogenate (40x).

The first known photographic images of *T. gondii* were published in 1923 by Josef Janku [5] taken from the retina of an infant later recognized to be suffering from congenital toxoplasmosis (the disease caused by *T. gondii* when the parasite is passed from mother to fetus). Tachyzoites, the crescent shaped form of *T. gondii* (Figure 1A), were described by Janku as "small, cylindrical, [and] bat-like cells in rosette form" associated with disrupted layers of cells in the retina with nuclei that were stained pale blue by hematoxylin. Colorful descriptions of specimens were common in the early 20^th^ century because while the publication of photographs to accompany manuscripts was becoming increasingly popular, the photographs were still printed in black and white. Tachyzoites have been stained and documented using a variety of methods. Splendore first observed them in wet preparations with a "yellow cytoplasm and a granular nucleus" [2] but also notes that they are seen more easily when using Giesma stain, which is specific for negatively charged phosphate groups on DNA. Tachyzoites are also easily visualized with hematoxylin (which stains nuclear proteins by forming violet colored complexes with metal ions) and eosin (a red acidic dye that stains basic proteins nonspecifically) [5] (Figure 1A-C), but stain poorly using Periodic acid-Schiff (PAS) [6], which oxidizes polysaccharides allowing them to react with Schiff reagent producing a pink color.

Light microscopy has also contributed to our knowledge of the behavior of tachyzoites. *T. gondii’s* discoverers noted that this form of the parasite reproduces by "bipartition" [1,2] (Figure 1B), though whether they made this observation in fixed tissue is unclear. It was later demonstrated by silver staining that tachyzoites reproduce by endogeny [7], which differs from mitosis in that the growth of daughter cells within the parent eventually consumes it.

A study showing that tissue cysts containing bradyzoites was also published by Janku [5]. Again, he reported that *T. gondii* was most strongly stained with haemotoxylin and eosin (H&E), but that staining with Giemsa, Mallory (a mixture of three dyes: acid fuchsin, aniline blue, and orange G, used to reveal red nuclei, pink cytoplasm and blue extracellular matrix), or Biondi (a mixture of aurantia, acid fuchsin and methyl-green to reveal pale cytoplasm and greenish chromatin) methods of staining also produced clear images. Bradyzoites (Figure 1C) within tissue cysts (Figure 1D) contain many granules of amylopectin, perhaps as a source of energy that is not present in tachyzoites, that stain red with PAS reagent [8]. This could make PAS a more specific stain for the presence of bradyzoites. The cyst wall is only slightly stained by PAS reagent, but heavily stained by Palmgren silver. Curiously, methenamine silver does not stain the cyst wall at all, suggesting that it is devoid of polysaccharides [8]. Although silver and PAS staining make it easier to distinguish a tissue cyst compared to Geimsa or H&E staining, it has been suggested that light microscopy is an inferior method for identifying cysts within tissue as they are easily confused with groups of tachyzoites or other parasites. A drawback of light microscopy that contributes to this confusion is that although a large increase in magnification is possible using visible light, resolution is limited. It has also been pointed out that it is unclear when the cyst wall is capable of appearing as silver positive, so more specific staining procedures could be used to identify a tissue cyst [6]. For example, it is now thought that Samuel Darling was the first to describe Toxoplasmosis in a human adult. However, at the time he diagnosed his patient with *Sarcosporidum*, which can easily be confused with a *T. gondii* tissue cyst when stained with H&E [9]. (Darling published illustrations with his original manuscript, not photographic images). Despite these reservations, the above staining methods were sufficient to determine the complete life cycle of *T. gondii*[10].

The 1934 invention of phase contrast microscopy [11] that earned Frederick Zernike a Nobel prize in 1953 [12] allowed the observation of cells and organelles on unstained, living specimens. In addition, developments including differential interference contrast (DIC), also called Nomarski Interference Contrast (NIC) [13], in the early 1950s allowed greater contrast in transparent specimens without the bright halo seen using phase contrast microscopy. Both phase contrast and DIC were used in conjunction with video microscopy to investigate proteins that are critical for parasite motility and cell invasion. This technique revealed that tachyzoites are highly motile despite their lack of flagella or cilia that other protozoa utilize [14] and they achieve this motility using an actin-myosin containing 'glideosome’ in the parasite’s inner membrane complex [15,16]. Parasites do not gain entry into the host cell by simple phagocytosis but by actively penetrating the plasma membrane [17] involving the coordinated secretion of microneme and rhoptry proteins that form the 'glideosome’ and 'moving junction’ respectively [18-20]. Although these experiments require the genetic manipulation of the parasite to target the protein of interest, it is the ability to image the resulting behavior that really tests the function of these molecules. Another critical aspect of *Toxoplasma* biology revealed by DIC imaging was the formation of the parasitophorous vacuole (PV) [21]. Here imaging was combined simultaneously with patch clamping cells to monitor changes in cell membrane electrical capacitance as the parasite attached; invaded and 'pinched’ off host cell membrane to form its intracellular niche.

### Electron microscopy

**Figure 2:**
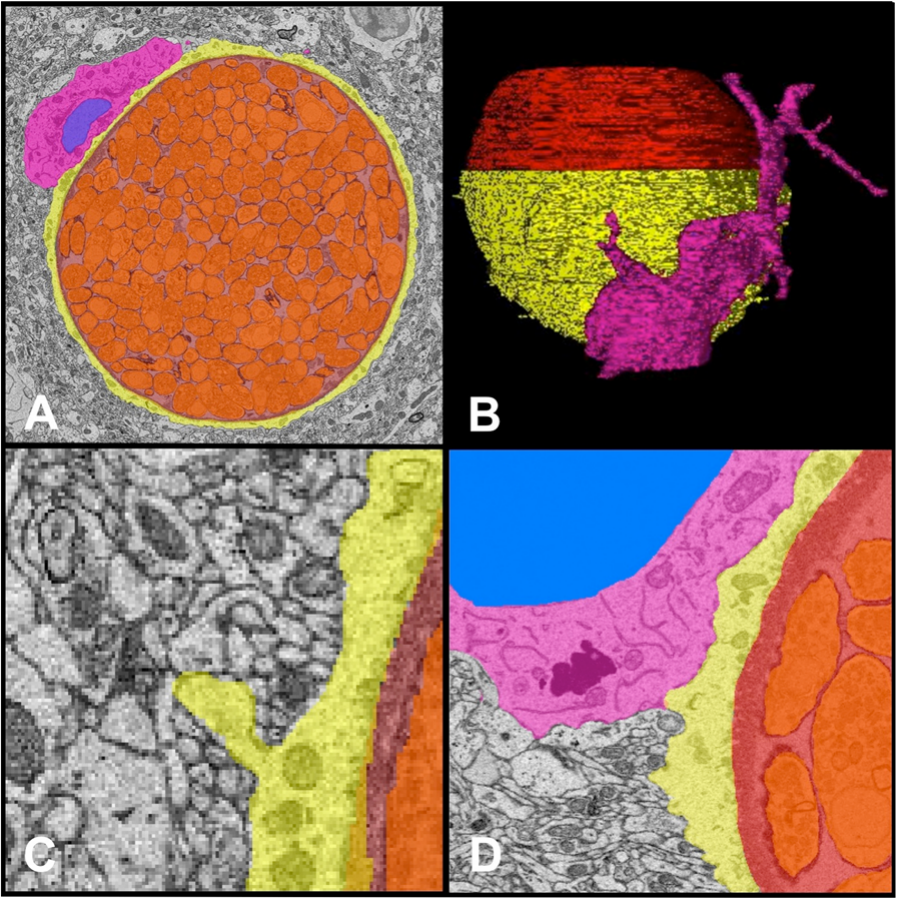
**Tissue cyst visualized by EM. A**. Myeloid cell (pink) intimately associated with a neuron (yellow), infected and stretched thin by a large cyst (red) containing hundreds of bradyzoites (orange). **B**. 3D reconstruction of infected neuron pictured in **A**. **C**. Synaptic vesicles in the presynaptic neuron (grey) is evidence the infected neuron (yellow) is still functioning. **D**. Large lysosomal bodies, characteristic of myeloid cells (pink), are present in close association to the infected neuron (yellow).

To visualize subcellular structures and improve the resolution of images at very high magnification, electron beams replaced visible light to create a technique known as election microscopy (EM). Using this technique, magnifications of up to 10^6^× are possible, and a resolution of 50 pm has recently been achieved [22]. Two techniques, transmission and scanning electron microscopy arose from this advance. Both require that samples be fixed, and dehydrated or flash frozen to prevent formation of ice crystals and kept in a vacuum to prevent the electron beam scattering off molecules in the air rather than the object of interest. Additionally, samples can be stained with metals such as lead or gold to add contrast. Those destined to be imaged using transmission electron microscopy (TEM) (Figure 2) are embedded in Epon and sliced into ultrathin sections prior to staining so that they are partially transparent to the electron beam. Samples prepared for scanning electron microscopy (SEM) do not need to be ultrathin; the image is generated by electrons emitted from the sample surface providing fine detail of the surface structure of relatively large solid objects. TEM was first employed in *Toxoplasma* research in 1954 [23] to study the ultrastructural morphology of the tachyzoite and later, of each stage of *T. gondii’s* life cycle [6,10,24,25]. Of the forms of *T. gondii* found in intestinal epithelium of cats, only the later stages have been studied by TEM. After free tachyzoites, free bradyzoites or tissue cysts are ingested by a cat, merozoites form and initiate gamete formation [6]. TEM imaging demonstrated that female gamonts are spherical and contain both rough and smooth endoplasmic reticulum, micropores, several mitochondria, double-membraned vesicles that are thought to derive from a central nucleus and two types of wall-forming bodies [25]. Male gamonts have an ellipsoidal shape, but after division into microgametes, they become elongated with a pointed anterior end and two flagella that extend posteriorly, stemming from the basal bodies in the anterior end. Microgametes fertilize the female gamonts to form zygotes, each surrounded by a five-layered oocyst wall [25]. Outside the cat, the oocyst will sporulate, giving rise to four sporozoites, each with an ultrastructure very similar to the tachyzoite [25]. The use of TEM for fine structure analysis also revealed that the bradyzoite has few ultrastructural differences from the tachyzoite and reproduces by endogeny within the spherical wall of tissue cysts [26]. The cyst wall is thin, made of material from both the host cell and the parasite and can enclose as little as two or as many as hundreds of bradyzoites [27] (Figure 2A). In contrast, SEM helped visualize the ultrastructure of tachyzoite entry into the host cell [28,29]. Both techniques have provided a wealth of information about the shape and organization of the parasite’s membranes, organelles [6,30,31] and cytoskeleton [32], and the structural changes that take place during host cell invasion, tachyzoite endodyogeny [33,34], parasite egress from the host cell [35] and evasion of host cell defenses [36-39]. Organelles unique to the parasite have become the object of intense research in the hopes of understanding *T. gondii*’s singular biology and finding novel therapeutic targets to combat this ubiquitous parasite.

Recently advances in SEM technology (and possibly more importantly computer and data handling) have facilitated whole tissue sectioning and 3D reconstruction. Fixed tissue samples are serially sectioned and subjected to SEM, resulting in a tomogram of the tissue. Cells in this tissue can be traced through slices, and these traces are superimposed on each other in space to create a 3D object. These objects can be viewed from any angle in three dimensions, providing a more complete picture of a cell than that obtained through traditional electron microscopy. Furthermore, it is possible to trace sub-cellular structures such as the nuclei or organelles of a cell and generate a 3D model of the sub-cellular environment. This provides a complete spatial picture of a cell and its components, which is unattainable with most other imaging methods. This has provided further beautiful images of the organization and polarity of rhoptry and microneme proteins from *in vitro* cultured tachyzoites [40,41]. In addition, we have recently imaged parasite and host cell interactions from infected brain tissue (Figure 2A-C). The reconstruction allows a clear image of the morphology of a myeloid cell interacting with a cyst-containing neuron (Figure 2A and B). This image supports the neuronal intracellular location of cysts and also suggests that these neurons are still functioning (Figure 2C). The idea that this is a silent process, however, does not seem to be supported and instead is supportive of a role for macrophages and T cells in controlling cyst burden (Figure 2D) [42].

However, unlike light miscroscopy, TEM and SEM studies are difficult to perform. Ice crystals commonly cause artifacts that can be difficult to distinguish from genuine structural features and considerable training is required to identify them. Also, metals used to stain samples are costly. Although the development of bench top SEM and TEM has made this technology more accessible, they still cannot completely replace full size instruments in terms of resolution and capacity for sample size. These full sized instruments need to be stored in their own rooms due to their size and sensitivity to magnetic fields. Moreover, while EM does provide phenomenal resolution it still requires fixing of tissues, thus artifacts associated with cross-linking of proteins could appear. In addition there are limitations on what we can gain about parasite behavior and interactions with its host. Further, although 3D electron microscopy can be a powerful imaging tool, it can be cost prohibitive both in terms of the cost of generating EM serial slices, and in the cost of buying hardware that can process three dimensional images efficiently. There is also a certain degree of luck involved in finding the needle in the haystack – finding a 15-20 μm cyst in a tissue that is approximately 12000 μm^3^ takes a degree of targeting!

### Fluorescence and bioluminescence

**Figure 3:**
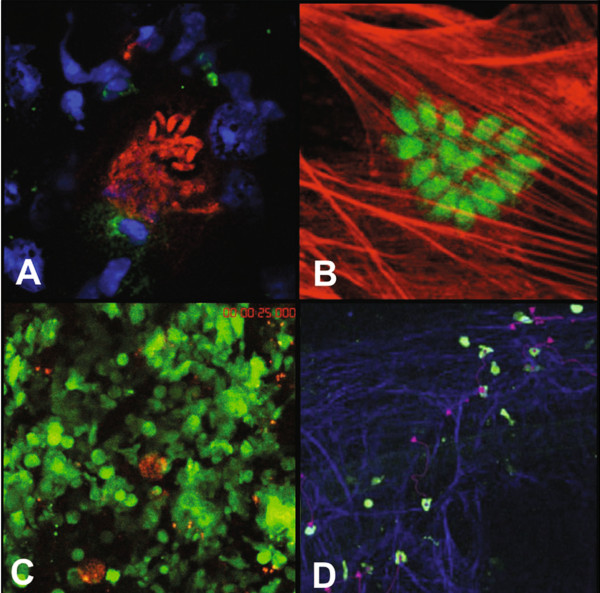
**Imaging *T. gondii *with parasite-specific antibodies, reporter parasites, and reporter hosts. A**. Anti-*Toxoplasma* antibodies used to visualize bradyzoites (red) escaping from a cyst that is next to a CD8+ T cell (green) in the brain of an infected mouse. Nuclei are stained with DAPI (blue). (40x) **B**. Pru-GFP, a *T. gondii* strain engineered to fluoresce green, is shown after having invaded a cultured human foreskin fibroblast (HFF) (actin stained in red) (63x) **C**. Me49-RFP cysts and GFP + leukocytes (green) are visualized in live brain tissue in a DPE-GFP mouse using two-photon microscopy. **D**. OTI-GFP T cells (green) migrating along a fibrous network (blue) in a live brain infected with Pru-OVA, visualized by two-photon microscopy.

The discovery of Green Fluorescent Protein (GFP) in 1978 [43], its many colored derivatives, and the increasing ease of publishing in color, led to an imaging revolution from which the field of *T. gondii* research benefitted immensely. Individual proteins, both on the parasite itself and in the host (Figure 3A) can be visualized in fixed tissue through the binding of fluorescently labeled antibodies raised against the proteins of interest. Although this technique is restricted to proteins that can be purified without contaminants for the production of antibodies and long term imaging of samples is subject to photobleaching, there are a variety of applications for this method. The use of FITC conjugated antibodies raised against host-cell cytoskeletal components demonstrated that while phagocytosis of the parasite required cytokeletal remodeling, active invasion and formation of the PV did not [17]. Further, imaging established that while phagocytosed tachyzoites can be killed when the phagosome fuses with the lysosome, tachyzoites can escape this fate by invading the cell from the phagosome, as the parasitophorous vacuole does not fuse with the lysosome [17]. Additionally, the use of fluorescein labeled monoclonal antibodies specific to bradyzoites determined that stage conversion between tachyzoite and bradyzoite was asynchronous and that tachyzoites and bradyzoites can coexist in the same parsitophorous vacuole [44]. Thus, it was proven that stage conversion is not a linear progression, but a dynamic process.

The parasite’s amenability to genetic manipulation gave way to a new approach, leading to the production of *T. gondii* strains stably expressing GFP [45] and other reporter proteins (Figure 3B) beginning in 1998. Suddenly, mere snap shots of the parasite’s biology were not the only tool available to researchers. For instance, stage specific expression of both red and green fluorescent fusion proteins allowed for stage conversion to be visualized in live cells both *in vitro* and *in vivo* using both fluorescence and confocal microscopy [46]. Real time imaging can also be used to witness the parasite’s attempt to subvert the innate immune system. For instance, natural-killer cells are known to kill cells infected with *T. gondii. Ex vivo* confocal imaging of interactions between dendritic cells (DCs) and natural-killer (NK) cells were shown to be prolonged, however, it was unexpected to watch parasites escape from the dying DCs and into the NK cells [47].

Fluorescent fusions to specific rhoptry and microneme proteins allowed visualization of protein trafficking within the parasite and progressive deletion analysis of those same proteins indicated which protein sequences are essential for the protein to be trafficked correctly [48]. Fluorescent tracers unattached to proteins also began to be used. For example, the selectivity of the parasitophorous vacuole membrane was demonstrated when it was shown that it excludes dyes such as Lucifer yellow from coming in contact with parasites within the vacuole [49].

The parasite’s virulence and ability to cause systemic infection can be visualized in real time using parasites engineered to express luciferase and hosts injected with the enzyme’s substrate, luciferin [50,51]. After interperitoneal injection, luciferin distributes quickly and without regard for any blood-tissue barrier [52]. Images of luciferase activity are produced by a charged-coupled device (CCD) camera and overlayed onto a picture of the host itself for reference. Luciferase activity can then be quantified by measuring the total number of photons emitted per second. This technique is unique as it is noninvasive and allows the dissemination, virulence, and location of the parasite to be imaged in the same animal over the course of infection.

Luciferase expressing parasites were first used *in vivo* to demonstrate that DCs infected with *T. gondii* are hypermotile and that DC infection augments dissemination of the parasite [53]. However, there are limitations to this technique. The intensity of the light emitted from luciferase activity decreases due to dark pigments in certain organs and fur so studying parasite dissemination in mouse strains engineered on a C57BL/6 background (a black mouse) requires that the mouse be shaved so the emitted signal can be seen. Further, a minimum number of parasites need to be present before a signal can be detected, therefore, this technique does not offer the resolution required to view parasites on a single cellular level. The study of parasite infectivity is also currently limited to small animals as bioluminescent signal cannot currently be detected in very deep tissues [52].

**Figure 4:**
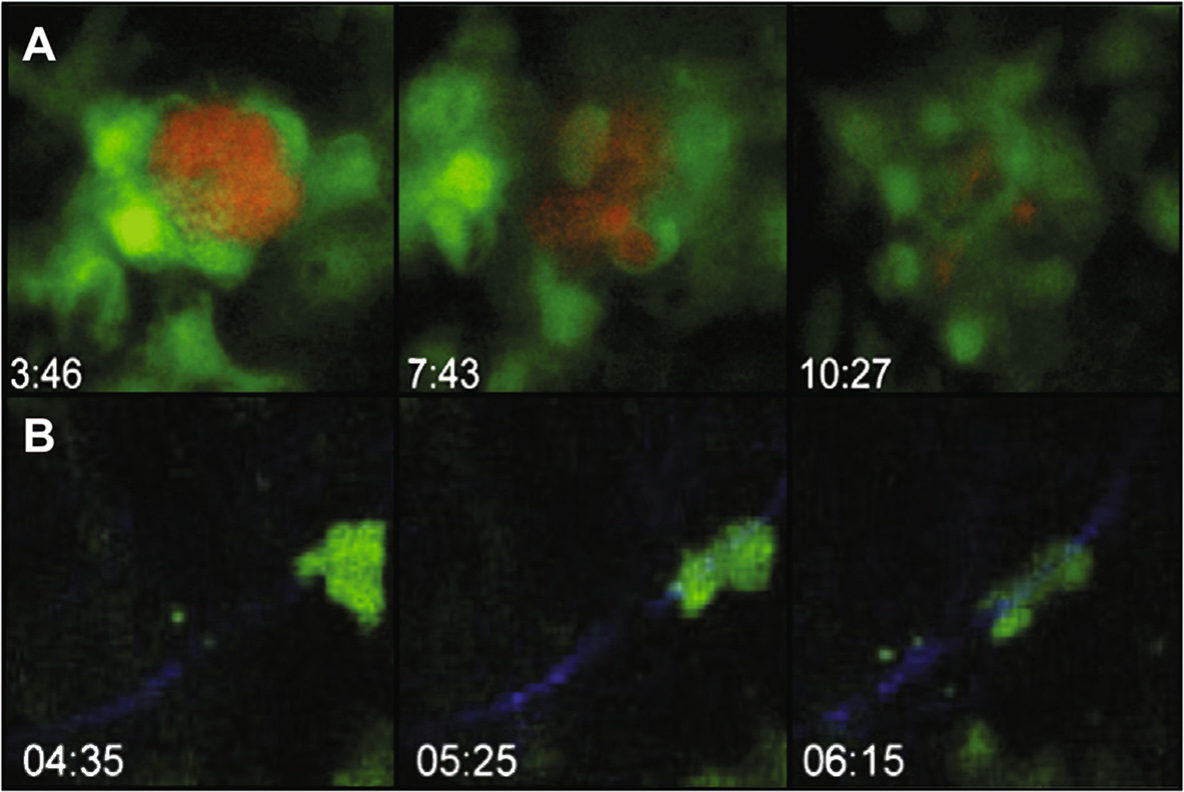
**Movies record cell behavior in response to parasite infection. A**. Three frames from a movie recording fluorescein labeled macrophage mediated destruction of an RFP + cyst *in vitro*. **B**. Three frames from a movie depicting GFP + T cell motility along a fiber visualized by second harmonic generation in a living brain.

As fluorescent tools started to proliferate, reporter parasites began to be used in combination with reporter hosts enabling the visualization not only of the parasite, but also of the responding immune cell environment (Figure 3C and D). For example, bone marrow expressing GFP from transgenic mice was transferred to irradiated wild type mice. These mice were then infected with parasites expressing red fluorescent protein. This experiment showed that CD11b + CD11c + cells were instrumental for *T. gondii* to gain access to the brain. Not only did infected cells exhibit increased extravasation into the brain, but curiously they were only populated with one parasite per cell [54]. More recently, the use of CellTracker labeled wild type and acidic mammalian chitinase (AMCase)-/- macrophages co-cultured with RFP expressing parasites demonstrated that chitinase secreted by alternatively activated macrophages is responsible for cyst lysis and may be the effector mechanism for the decrease in cyst burden seen in wild type mice over time [42] (Figure 4A). These unexpected results might have gone unnoticed had the ability to image live cells using fluorescent proteins not been developed.

### Multiphoton microscopy

The advent of multiphoton imaging in live cells beginning in 1990 [55] has presented researchers the opportunity to achieve a long sought-after goal: imaging dynamic interactions between the parasite and its host-cell in real time. Multiphoton microscopy uses low energy photons in short pulses to image at greater depths within tissue, reduce light scattering and minimize photobleaching to allow for long term visualization of labeled cells. With the application of reporter hosts and parasites, the improvement of surgical techniques to access particular tissues and this new technology, researchers can now image parasite and host-cell behavior within live tissue for up to several hours.

One of the first studies using this technology during infection focused on neutrophil behavior in the lymph node during acute *T. gondii* infection. The authors were able to show that during infection, neutrophils can enter the lymph node via both blood and lymphatic vessels and contain proportionally more parasites than both macrophages and DCs very early during infection. Using both LysM^GFP^ mice (where GFP expression is driven by the promoter for Lysozyme M) and RFP parasites it was also demonstrated that swarms of neutrophils are recruited to the subcapsular sinus in the lymph node in response to acute infection. The dynamics of two types of swarms, small and transient or large and persistent were observed that coincided with locations of parasite egress. Neutrophils infected with parasites move significantly slower than their uninfected counterparts and were often observed at the center of swarms. In contrast, uninfected neutrophils migrate in a quick and directed manner following parallel paths to join either transient or persistent swarms and these swarms lead to the reduction of subcapsular sinus macrophages in the lymph node [56].

DC interactions with T cells in the lymph node are also the subject of study during early infection with *T. gondii*. CD8+ T cell priming in the sub-capsular region was demonstrated to occur early during initial infection by adoptively transferring naïve OT1^GFP^ T cells into wild type recipients and infecting them. DCs were shown to be required for T cell expansion and in the presence of enough antigen, the velocity of CD8+ T cells decreased and the amount of interaction between T cells and DCs tapered off over time. It was also shown that the organization of the network of reticular fibers along which cells migrate in the lymph node changed over time. This network is visualized by detecting second harmonic signals generated by non-centrosymmetric structures such as collagen. Second harmonic signals can be detected in the 457-487 nm range after excitation with 930 nm light. Upon infection, the volume of this network increases, coinciding with the decrease in well-defined B cell follicles and T cell zones [57].

New details of parasite and cell behavior in the brain during chronic infection have been revealed by the multiphoton technique. Using OT1^CFP^ T cells with RFP reporter parasites and GFP labeled CD11b + antigen presenting cells (APCs), one study demonstrated that antigen specific CD8+ T cells were recruited to the brains of chronically infected mice and remained there as long as antigen was present [58]. Another study found that a population of T cells clusters and arrests near infected cells (Figure 3C). The authors of this study went on to show the upregulation of a fibrous network in the brain upon infection visualized by second harmonic signals (Figure 3D), similar to what is seen in the lymph node. Highly motile GFP expressing CD8 T cells migrated along this network (Figure 4B) and were targeted toward areas of parasite replication. It is unclear what this network is made of in the brain, as infection does not lead to an increased expression of collagen as it does in the lymph node. Further, this study showed that the well-known phenomenon of astrocyte activation during chronic *T. gondii* infection [59] involved astrocytic swelling [60]. The ability to record the dynamic nature of these interactions in the brain has also led to the ability to more reliably quantify cell behavior *in vivo*, rather than relying on a descriptive image.

Despite all the advantages of multiphoton microscopy, the equipment required to perform these assays is still cumbersome and extremely costly. Another drawback to this technique is that imaging still cannot reach the depths that some researchers would like so multiphoton imaging is still an invasive procedure because the organs of interest still need to be surgically exposed.

## Conclusions

Since *T. gondii* was discovered 100 years ago, imaging technology has considerably advanced. It has provided us with an understanding of *T. gondii’s* complex life cycle and dynamic interactions within its host. The imaging techniques reviewed here ranged from the easy and inexpensive to technically challenging and costly. The use of imaging in general is still very descriptive but with complementary data from techniques such as flow cytometry and the advent of multiphoton microscopy, *in vivo* dynamics are becoming increasingly quantitative by allowing researchers to calculate the parameters of parasite and host cell behaviors. Advances in imaging technology are continuously being made. The ability to view details of an entire organ in high resolution will likely lend further advances in our understanding of *T. gondii* behavior, including its entry into specialized areas like the brain. An exciting new application of this technology is serial two-photon (STP) tomography, where an entire brain can be imaged in an automated manner by combining two-photon microscopy with tissue sectioning [61].

The next hurdles imaging technology needs to cross are cost and accessibility. An interesting step in this direction has been the creation of a miniaturized fluorescent microscope [62]. This technology is currently being applied to *in vivo* imaging of non-anesthetized mice. While it still needs a computer to operate and store data, it is intriguing to imagine any type of microscope becoming so small that a researcher could put it in his or her pocket and travel with it. One can also imagine a time when this technology can be used to image a single cellular interaction deep within the tissue of a live and active mouse rather than an anesthetized mouse.

With new imaging technologies and applications developing every day, the future of *T. gondii* research is bright, not just for the images we are able to produce, but also for the complex and intriguing questions these advances will allow us to answer.

## Abbreviations

*T. gondii*: *Toxoplasma gondii*; H&E: Hematoxylin and eosin; PAS: Periodic acid-Schiff; EM: Electron microscopy; TEM: Transmission electron microscopy; SEM: Scanning electron microscopy; GFP: Green fluorescent protein; FITC: Fluorescein isothiocyanate; DCs: Dendritic cells; NK: Natural killer; CCD: Charged coupled device; RFP: Red fluorescent protein; STP: Scanning two-photon.

## Competing interests

The authors declare that they have no competing interests.

## Authors’ contributions

KEM and EHW wrote the review. KEM generated images of H&E stained and fluorescently labeled parasites and helped generate 3D EM images. EHW edited and discussed the review and generated images of activated astrocytes, migrating T cells and unstained cysts. Both authors read and approved the final version of the manuscript.
